# Review of the Immune Checkpoint Inhibitors in the Context of Cancer Treatment

**DOI:** 10.3390/jcm12134301

**Published:** 2023-06-27

**Authors:** Norah A. Alturki

**Affiliations:** Clinical Laboratory Science Department, College of Applied Medical Sciences, King Saud University, Riyadh 11433, Saudi Arabia; noalturki@ksu.edu.sa

**Keywords:** immune checkpoint inhibitors, PD-1, PD-L1, CTLA-4, cancer treatment, neoantigens

## Abstract

Checkpoint proteins are an integral part of the immune system and are used by the tumor cells to evade immune response, which helps them grow uncontrollably. By blocking these proteins, immune checkpoint inhibitors can restore the capability of the immune system to attack cancer cells and stop their growth. These findings are backed by adequate clinical trial data and presently, several FDA-approved immune checkpoint inhibitors exist in the market for treating various types of cancers, including melanoma, hepatocellular, endometrial, lung, kidney and others. Their mode of action is inhibition by targeting the checkpoint proteins CTLA-4, PD-1, PD-L1, etc. They can be used alone as well as in amalgamation with other cancer treatments, like surgery, radiation or chemotherapy. Since these drugs target only specific immune system proteins, their side effects are reduced in comparison with the traditional chemotherapy drugs, but may still cause a few affects like fatigue, skin rashes, and fever. In rare cases, these inhibitors are known to have caused more serious side effects, such as cardiotoxicity, and inflammation in the intestines or lungs. Herein, we provide an overview of these inhibitors and their role as biomarkers, immune-related adverse outcomes and clinical studies in the treatment of various cancers, as well as present some future perspectives.

## 1. Introduction

Microenvironment of the tumor is monitored and regulated by immune surveillance through innate and adaptive immune systems [1]. Antigen-presenting cells have a fundamental role in this surveillance, as they identify and present tumor neoantigens to naive T-cells [2]. When exposed to the antigen, naive T-cells multiply and become activated to initiate an anti-tumor immune response [3]. This reaction is controlled by both stimulatory and inhibitory signaling molecules [4,5]. The inhibitory signals are mediated through immune checkpoints, such as B and T lymphocyte attenuator (BTLA), programmed death protein 1 (PD-1) and cytotoxic T lymphocyte-associated protein 4 (CTLA4) [6,7]. CTLA-4 and PD-1 exist on the exterior of T-cells and can stop their activation [8]. CTLA-4 is also found on Treg cells, and aids in immune suppression. PD-1 is also found on the exterior of B and other immune response cells [9]. When PD-1 binds to its partner proteins, it can also stop the T-cell response [8]. However, cancer cells within the tumor microenvironment can escape the anti-tumor influences by stimulating these checkpoints through CTLA4 or PD-1/PD-L1 expression enhancement. They also attempt this by suppressing antigen presentation [10,11].

Overexpression of these checkpoints on immune cellular surface can limit the capacity of the immune system to identify and attack cancerous cells. This permits the cancer cells to escape scrutiny and grow uncontrollably [12,13]. This is common in cancer cells and can be a mechanism of resistance to immunotherapy [14]. Additionally, the unique proteins or neoantigens, generated by genetic mutations in cancer cells are often taken as foreign by the immune system and can serve as targets for the immune response against cancer [15]. However, in some cases, the immune system may not properly distinguish neoantigens as foreign, causing the cancer cells to evade immune surveillance and grow. This can occur when the immune system is suppressed by checkpoint proteins, such as CTLA-4 and PD-1/PD-L1 [16]. Checkpoint proteins limit the action of immune cells by tethering specific ligands on the surface of cancer cells. This interaction conveys a signal to the immune cells to decrease their response, allowing the cancer cells to escape immune attack [17,18]. By blocking checkpoint proteins, immune checkpoint inhibitors (ICIs) can augment the impact of the immune system to counter cancer cells more effectively [19]. Thus, neoantigens generated by genetic mutations in cancer cells can serve as targets for immune-mediated tumor control. However, immune response activity may be limited by checkpoint proteins, and blocking these checkpoint proteins with ICIs can destroy carcinogenic cells and improve the outcomes of immunotherapy [20]. Overexpression of immune checkpoints can also be targeted by these drugs to block their pathway and boost the immune system to spot and attack unchecked proliferating cells.

This review is focused on the ICIs and their role in cancer treatment. The history of ICIs in immunotherapy dates back to the 1890s [21] but their use for cancer cure only dates back to the 1990s when researchers first identified the importance of immune checkpoints in regulating the immune response [22,23]. The first anti-cancer ICIs were developed in the early 2000s but received regulatory endorsement for skin cancer treatment only after 2010 [24]. Since then, ICIs have become a significant part of cancer management and they have had a noteworthy impact on patient outcomes in melanoma [25], lung cancer [26], hepatocellular carcinoma [27], head and neck cancer [28], ovarian cancer [29], renal cell carcinoma [30] and many others. The use of these drugs has led to long-lasting treatment responses and even cures in advanced-stage cancer patients.

## 2. Methodology

To provide a broad overview of the existing literature, we conducted a narrative review of the ICIs used for cancer treatment. We used PubMed, Scopus, and Google Scholar for searching the literature. A list of keywords and phrases, e.g., ‘immune checkpoint inhibitors’; ‘ICIs’; ‘ICI clinical trials’; ‘Cancer’ AND ‘immune checkpoint inhibitors’; ‘Cancer’ AND ‘ICIs’, etc., were used to search the databases. We did not add a filter for a specific year, or the study type, to include all the data available related to ICIs in cancer treatment. Exclusion criteria included articles written in a language other than English and studies providing insufficient information in the abstract to evaluate their relevance. For this purpose, the titles and abstracts of the retrieved articles were reviewed manually to assess their relevance to the topic and the studies not meeting the context of ‘ICI in cancer treatment’ were excluded. The included studies were read and analyzed. Notes on study design, methodology, or other details of importance were written down. Collected information was then structured to present a comprehensive overview of the topic. The Clinical Trials database (https://clinicaltrials.gov/; accessed on 22 February 2023) was also consulted to obtain the latest information on cancer treatment trials using ICIs.

## 3. Working Principle of ICIs

ICIs work by modulating the immune cell system, overexpressing themselves, and eradicating the immune inhibition directive of the cancerous cells [31]. The immune system, vital for resistance against infections and neoplasms, may become suppressed and fail to properly recognize and eliminate cancer cells in certain circumstances. ICIs address this issue by regulating the immune checkpoints and restoring or enhancing the function of the immune system for tumor suppression or elimination [32]. ICIs are often used in amalgamation with other treatment procedures, like surgery, chemotherapy, or radiation therapy to elicit an efficacious anti-tumor response and enhance the effectiveness of the cancer treatment [33]. The ultimate objective of ICIs is to harness the potent effector functions of the immune system for combating cancer, thereby improving patient outcomes.

**Figure 1:**
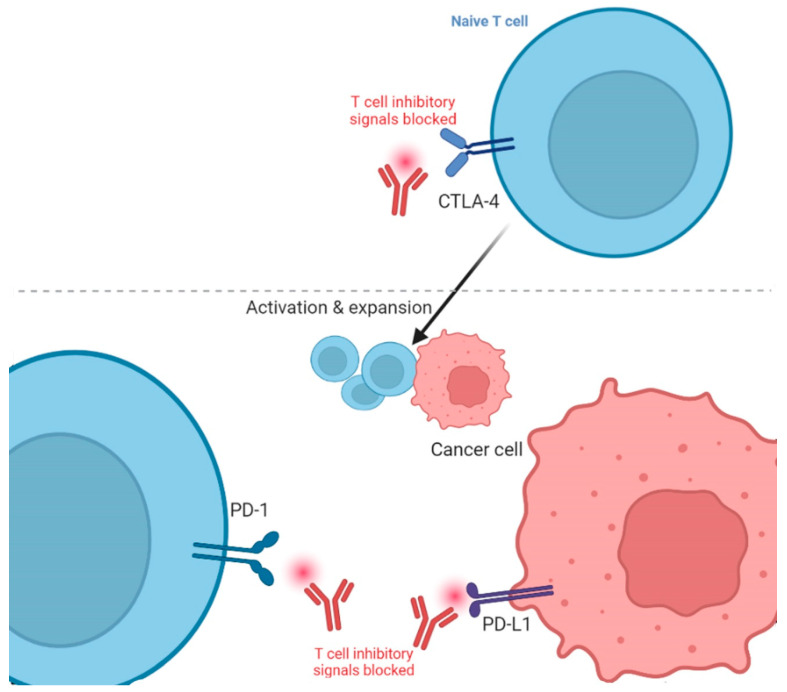
Blockade of cytotoxic T lymphocyte-associated protein 4 (CTLA-4) and programmed death protein 1/Ligand 1 (PD-1/L1) in cancer immunotherapy. The figure is adapted from Ribas, 2012 [39] and created using icons from biorender.com (accessed 12 February 2023).

The two main types of ICIs are CTLA-4 and PD-1/programmed cell death ligand 1 (PD-L1) inhibitors [34]. CTLA-4 inhibitors target the CTLA-4 protein, which is present in the T-cells and acts as a brake on the immune system [9]. By obstructing the CTLA-4, these drugs allow T-cells to become more active and kill cancerous cells. PD-1/PD-L1 inhibitors block the relevant PD-L1 ligand expressed in the cancer cells (Figure 1) [35]. By obstructing this complex, these drugs inhibit the tumor from immune escape and sanction T-cells to eliminate cancer cells. Thus, overexpression of immune checkpoints can lead to decreased effectiveness of the immune system in distinguishing and killing cancer cells. Pursuing checkpoint proteins with ICIs can enrich the anti-cancer immune response and improve the outcomes of immunotherapy. The first ICI against CTLA-4 was named Ipilimumab, and got FDA approval for melanoma treatment in 2011 [36], while the first PD-L1 ICI against melanoma was Pembrolizumab and got FDA approval in 2014 [37]. It is effective for melanoma that progresses even after administering Ipilimumab [38].

## 4. Immune-Related Adverse Responses to ICIs

The immune response to ICIs can vary but is different from the response of classical chemotherapy. Many patients experience side effects, such as fatigue, skin rashes, and colitis (inflammation of the colon) [40]. Some patients may experience a strong reaction, called an immune-related adverse event (irAE). irAEs can severely impact various organs, such as the gastrointestinal tract, skin, liver, endocrine glands, myocarditis, and others [41,42]. The frequency of side effects from irAEs due to ICIs depends on the type of drug and the patient’s specific health characteristics. The estimated chance of a fatal side effect from these drugs is between 0.3% and 1.3% [43]. Fatal or adverse side effects from immunotherapy drugs usually occur early in treatment and can be serious. However, this risk is still lower compared to other treatments like chemotherapy [44] or stem cell transplantation [45]. The type of side effect can also vary based on the combination of drugs used. For example, death from colon inflammation is more frequent in patients receiving anti-CTLA-4 drugs [46,47,48], while death from lung inflammation occurs more in patients receiving anti-PD-1 or anti-PD-L1 drugs [43,49,50].

irAEs due to anti-CTLA-4 antibodies’ administration occur in 60% of treated patients, with various grades. Among these, 10–30% experience serious (grade 3–4) irAEs [43]. The risk of irAEs is dose-dependent, with higher doses being associated with a higher prevalence of adverse events [51]. The bulk of grade ≥ 3 irAEs ensue in 8–12 weeks of drug usage. Skin rash onsets at the earliest, while diarrhea and/or colitis are the most frequent irAEs caused due to the administration of anti-CTLA-4 antibodies [43,52,53,54]. Other toxicities include endocrinopathies, hepatotoxicity, and rare toxicities, such as neuropathies, autoimmune thrombocytopenia, and Stevens–Johnson-like syndromes [55]. Neurological irAEs occur in 3.8% of patients being administered with anti-CTLA-4 antibodies, while grade ≥ 3 adverse events occur in less than 1% of patients [43,56].

Compared to anti-CTLA-4 antibodies, anti-PD-1 antibodies are associated with less frequent irAEs [49]. Most Anti-PD-1-related irAEs arise in the initial 6 months of drug use [43]. Common effects (occurring in less than a quarter (5–20%) of patients) include rash, fatigue, arthralgia, headache, pruritus, diarrhea, colitis, pneumonitis, hepatitis, and endocrinopathies [57]. Only around 10% of patients using anti-PD-1 drugs experience irAEs of grade ≥ 3, compared to up to 30% in the case of anti-CTLA-4 antibodies [43]. Neurological irAEs occur in 2.9% of patients receiving anti-PD1 treatment [58]. However, skin, hepatic, and pulmonary-related irAEs are more frequent in the case of anti-PD1 antibody administration than anti-CTLA4 antibodies, it is the opposite for thyroid and lower digestive tract irAEs, such as colitis [43].

Initially, autoimmune disorders were excluded from irAEs but studies suggest that ICIs are non-toxic, manageable, or bearable for people with autoimmune diseases or associated symptoms who are also suffering from cancers [59,60]. Only a minority of patients have been reported to experience the exacerbation of previous autoimmune diseases. The ICIs may be administered safely to around 50–70% of the patient cohort with a preceding autoimmune disease [59]. The incidence of grade ≥ 3 irAEs in cancer patients is reported to be 1.58% in a meta-analysis [61], while in ICI clinical trials that excluded cancer patients with autoimmune diseases, the grade ≥ 3 irAEs ranged from 7 to 15% [62,63,64]. It has been also suggested that targeted immunosuppression, comprising anti-PD-1 antibodies in conjunction with antibodies aimed against selected inflammatory mediators can avert the aggravation of autoimmune diseases and this can be carried out without disrupting the efficiency of anti-PD-1 drugs [65,66]. Management, monitoring, and discontinuation of the drugs could be attempted in the case of irAEs.

## 5. ICI Biomarkers

Immunotherapeutic biomarkers vary from other cancer treatments as they are continuous and change over time, influenced by multiple factors [34]. Their use with chemotherapy makes the study of biomarkers more complex. Markers envisaging response and resistance to ICI are labeled positive and negative predictive biomarkers, respectively [67]. In addition to these, toxicity prediction is deliberated by side effect biomarkers [68]. Common positive predictive biomarkers in ICI treatment include tumor mutational burden (TMB) study [69], T-cell filtration [70], PD-L1 expression analysis [71], etc. Enhanced PD-L1, TMB, and T-cell infiltration within a tumor correspond to a better ICI treatment response [38]. Neoantigen’s ability to activate T-cells dissimilarly to self-antigens can also be used as a predictive biomarker [72]. FDA-approved biomarkers include PD-L1, TMB, and microsatellite instability (MSI) for patient selection toward gaining therapy response [73]. Biomarkers allied with the instigation of the immune process may be used for predicting the likelihood of a patient benefiting from any potential immunotherapy, for instance, TMB. High TMB is associated with an increased likelihood of detection by the immune system and thus elimination of cancer cells [74]. On the other hand, PD-L1 augments drug-specific effects [75].

PD-L1 was the maiden biomarker to be approved by the FDA in 2015 for non-small-cell lung cancer [76]. It has four FDA-approved testing methods to check the expression, with rabbit or mouse monoclonal antibodies [77]. It is now authorized as a companion indicative test for several tumor types and is currently in use as a predictive biomarker for ICIs, but work on other markers is being carried out due to its low diagnostic precision. Nonetheless, it has shown good results in predicting survival for some cancers. The second predictive biomarker to get FDA approval was MSI/DNA mismatch repair system (MSI/dMMR) biomarker in 2017 [78]. Tumors with malfunctioning dMMR accumulate numerous mutations across the genome and microsatellite regions being prone to these errors lead to MSI [79]. Non-MSI regions have an increased rate of mutations in tumors with dysfunctional dMMR and thus have additional neoantigens in comparison to a tumor with functional MMR [67,80]. The higher presence of neoantigens causes an enhanced immune response, including infiltration by lymphocytes, memory T-cells, and T-helper 1 cells, making them more responsive to immunotherapy. As a result, MSI/dMMR or tumor infiltration index can be used as a positive predictive ICI treatment response biomarker. Immunohistochemistry-based tests, PCR, or sequencing can be used for getting information regarding this biomarker [81]. The third predictive marker approved by the FDA is TMB. It is a count of the aggregate non-synonymous somatic mutations of the tumor. Increased mutation burden in somatic exonic regions causes enhanced neoantigen production [82]. Some neoantigens are immunogenic and their recognition by the T-cells results in better anti-tumor immune retort and sensitivity to ICI treatment. TMB can be tested via WES and NGS panels but is more challenging to study than PD-1 and MSI. Its estimation can be impacted by the tumor type, tissue type, sequencing parameters, etc. [67].

The response rate to immunotherapy is unpredictable via these markers and varies widely, with some patients with low or absent tumor expression for these markers still displaying a good response, and vice versa. This uncertain predictability highlights the need for other biomarkers in determining the response to immunotherapy. Gene signature predictive biomarkers, including T-cell inflamed gene expression profile (GEP), melanocytic plasticity signature (MPS), T-cell dysfunction and exclusion gene signature (TIDE), and B-cell focused gene signature have also been explored for ICI therapy [68]. High expression of GEP and TIDE has shown better patient survival statistics compared to TMB or PD-L1 in ICI therapies [83,84,85]. Low MPS has demonstrated longer survival [86]. It has outperformed FDA-approved markers in experiments. The combination of gene markers (TMB and GEP; MPS and TIDE) is also of superior prognostic value compared to single gene predictors [67]. Apart from these, the richness of B cells in tumors is also associated with better ICI responsiveness and patient survival [87,88]. PTEN inactivation, POLE mutations, and common mutations of KRAS and STK11 have predictive value as well [89,90,91]. Overall, these markers denote a significant prospect for ICI response prognostics. The ultimate goal is to use these biomarkers to personalize treatment and improve outcomes for patients.

## 6. ICIs in the Laboratory or Pre-Clinical Studies

In biomedical research, preclinical studies are the initial stage of research that involve testing a new drug or therapy in non-human models, such as cell lines, laboratory animals, cells, or organoids, to evaluate the efficacy and safety of the treatment [92]. The selection of the model is determined by the specific research question and the type of therapy being tested. Preclinical studies are usually conducted before human clinical trials and help to provide important information about the potential benefits and risks of the treatment [93]. They can help researchers to determine the optimal dose, route of administration, and timing of treatment. In addition, preclinical studies offer valuable information about the mechanism of action of the treatment, as well as its potential side effects and toxicity [94].

Non-human models, such as cell lines, spheroids, organoids, and animals like mice, zebrafish, dogs, primates, particularly macaques, etc., have been used in preclinical studies to evaluate the efficacy and safety of ICI therapy [95]. Tumor-transplanted macaques have been demonstrated as a useful preclinical model for investigating T-cell tumor accretion and for studying the development of new immunotherapies [96]. Hutchins et al. conducted an anti-PD1 assessment in non-human primates and found that safety results were comparable in human clinical trials [97]. Ji et al. developed a macaque model for determining ICI-induced multi-organ irAEs, specifically myocarditis [98]. The toxicity of combination therapy has also been studied in macaques for anti-PD1 and anti-CTLA-4 for urinary tract and skin cancer, respectively [99]. This study helped infer the inflammation response to these ICIs.

Patient-derived xenografts (PDX) are a type of preclinical model in which tumor tissue is taken directly from a patient and transplanted into a mouse or another animal with a compromised immune system. The mice are then monitored for changes in tumor growth, metastasis, and overall survival. PDX models are particularly valuable because they preserve the genetic and molecular characteristics of the patient’s tumor, making them more representative of the patient’s disease. Zhao et al. demonstrated the use of PDX for evaluating drug benefits and side effects of anti-PD1 and anti-CTLA-4 antibodies for hepatocellular carcinoma [100]. Odunsi et al. demonstrated enhanced T-cell production under the combined impact of these antibodies in both cell lines and PDX for ovarian cancer [101]. Zebrafish xenografts have also been studied for anti-PD1 response against pancreatic cancer, revealing initiation of apoptosis and shrinkage in the tumor size [102]. Several cell line studies have been conducted to investigate the potential of anti-PD-1 therapy in different types of cancers, including melanoma [103], lung cancer [104], and bladder cancer [105]. These studies have shown that ICI combination therapy can enhance anti-tumor impact, leading to improved outcomes for patients.

Preclinical mice models may include humanized immune system (HIS) and genetically engineered mice for studying the impact of ICI against various cancers (Table 1). These models have proven valuable in studying human immune responses to ICI therapy for various cancers [106,107].

Apart from these, tumor organoids and spheroids are in vitro three-dimensional models of cancer that closely mimic the architecture and heterogeneity of tumors in vivo. These models provide an excellent platform to study the response of tumors to ICI therapy and to test potential therapeutic strategies. Kong et al. have previously studied anti-PD-1 response in rectal cancer using organoids [116]. Jenkins et al. have studied anti-PD-1 response in melanoma and colon cancer using tumor spheroids [118]. Similarly, studying anti-PD-1/PD-L1 response in chordoma patient organoids revealed that organoids could prove useful for even those patients that lack immunohistochemical PD-L1 expression [119].

## 7. ICI Clinical Trials

The results of several preclinical studies may not prove useful when directly extrapolated for humans and many treatments that show promising results in preclinical studies do not ultimately prove to be safe and effective in human trials. This is why clinical trials must also be conducted before marketing drugs. Usually four levels of clinical trials may be conducted to ensure the usefulness and safety of a drug. Phase 1 typically involves a small number of patients (usually 15–50 individuals). The primary objectives of phase 1 trials are to evaluate the safety and toxicity of the drug or treatment, define a dose range, and detect prospective side effects [120]. Phase 2 trials typically involve a larger number of patients (usually 100–300 individuals) and are conducted to appraise the efficacy of the drug or treatment in a particular set of patient population [121]. Phase 2 trials also provide supplementary evidence regarding the safety profile of the drug or treatment and can help determine optimal dosing [122], while phase 3 trials are the largest and the most comprehensive clinical trials, typically involving thousands of patients. Phase 3 trials can confirm the efficacy of the drug or treatment, compare it to existing treatments, and further evaluate its safety profile [123,124,125]. The results of phase 3 trials are often used by regulatory agencies to make decisions about the approval and marketing of the drug or treatment [126], while phase 4 mostly includes post-marketing analysis [127].

**Figure 2:**
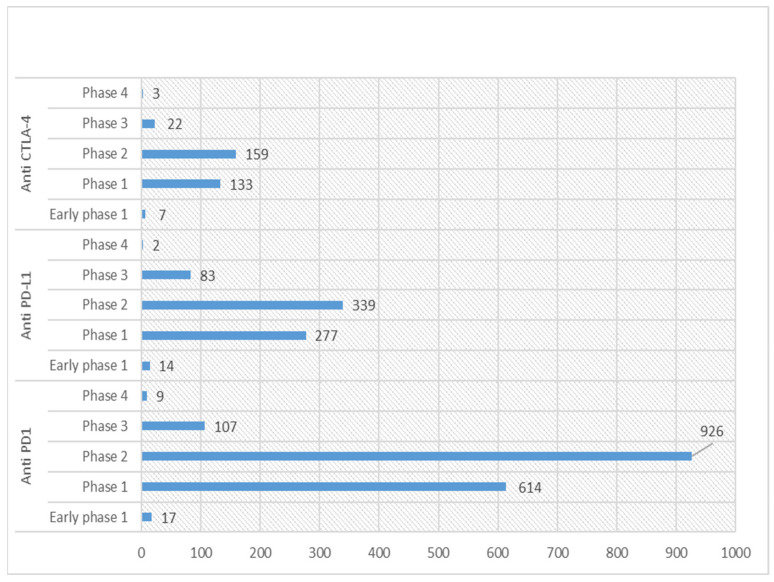
Graph showing statistics of various phases of clinical trials for anti-PD-1/L1 and CTLA-4 in US Clinical trials database (https://clinicaltrials.gov/; accessed on 22 February 2023).

Currently, the US clinical trials database (https://clinicaltrials.gov/; accessed on 22 February 2023) statistics show 853 active (recruiting or non-recruiting) clinical trials registered for anti-PD-1, 330 for anti-PD-L1 and 128 for anti-CTLA-4. Detailed statistics for various phases of these registered trials for ICIs are shown in Figure 2. Among these, around 11 phase 1 trials are ongoing for combination therapy (Table 2).

The results of the clinical trials for ICIs have been generally positive, with many patients showing significant improvements in survival and quality of life. Cancer immunotherapy was acknowledged as a ‘breakthrough of the year in 2013’ due to its substantial progress against cancer [128]. However, a lot more effort still needs to be undertaken for annotating interactions of the tumor with human immunome and its therapeutic interventions. Use of these drugs is associated with side effects, including irAEs, and further research is needed to better understand and manage these side effects [129]. The number of new ICI being registered are good but several of them are similar anti-PD-1/L1 antibodies and a large fraction of the trials is dedicated to T-cell modulation drugs [129]. Additionally, the patient recruitment rate needs to be improved. It is also important to note that not all drugs or treatments advance through all phases of clinical trials, and some may be discontinued at any point due to safety concerns or lack of efficacy. Some examples of these include termination of a basket trial for anti-CTLA-4 and PD-L1 combination therapy assessment in metastatic solid tumors (NCT03982173) and anti-CTLA-4 in advanced melanoma (NCT 01740401) due to non-encouraging results. An anti-PD-1 trial for refractory (NCT 03445533) and advanced melanoma (NCT02452424) was terminated due to lack of efficacy. Similar non-satisfactory results for phase 1/2 trials for anti-PD-L1 therapy against advanced melanoma and other solid tumors also resulted in the termination of trials.

## 8. Future Perspective

ICIs work in conjunction with the immune system and demonstrate an important stride toward cancer treatment. They help enhance and amplify the body’s own immune response to cancer, leading to an improved anti-tumor response. These drugs have revolutionized the way cancer is treated, and they have the potential to continue having a significant impact on patient outcomes in the future. However, like all cancer treatments, ICIs can have side effects, including irAEs, such as colitis, hepatitis, and skin reactions. These side effects can usually be managed with prompt recognition and treatment. The future of ICIs therefore holds a lot of promise for the treatment of cancer.

However, despite this success of ICIs in treating cancers, there are cases where they may not be as effective or cancers may resist ICIs [130,131] and require alternative therapeutic approaches. This includes cancers with low TMB, low PD-L1 expression [132,133], immunosuppressive tumor microenvironment or alternative immune evasion mechanism [134]. Cancers with low TMB, such as certain types of breast and prostate cancers, may not have enough neoantigens (antigens derived from tumor-specific mutations) to provoke a robust immune response [135], while a cancer with low PD-L1 expression cannot respond well to PD-1/PD-L1 inhibitors. In tumors that create an immunosuppressive microenvironment, the effectiveness of ICIs is hindered due to the presence of regulatory T cells, myeloid-derived suppressor cells, and other immune suppressive factors [136].

In such cases, targeted, combination, adoptive or vaccine-based therapies maybe useful. Combining ICIs with other treatments, such as chemotherapy [137], radiotherapy, targeted therapies, or other immunotherapies, may enhance the overall response [33]. It may also include targeting a dyad or triad of ICIs. Combination treatment with ICIs and multiple therapeutics is an active area of research and clinical development in the field of cancer immunotherapy [138]. The goal is to synergize different mechanisms of action, enhance the overall anti-tumor immune response in order to overcome resistance and improve patient outcomes [139]. This has been reviewed in detail elsewhere [140,141,142,143,144].

The focus of this review was CTLA-4, PD-1, PD-L1 but there are several promising targets for ICIs beyond them. These include Lymphocyte-activation gene 3 (LAG-3), T-cell immunoglobulin and mucin-domain containing-3 (TIM-3), T-cell immunoreceptor with Ig and ITIM domains (TIGIT), V-domain Ig suppressor of T cell activation (VISTA), and B7 homolog 3 (B7-H3) inducible T cell costimulatory (ICOS) [144], Fibrinogen-like protein 1 (FGL1) [145], B and T lymphocyte attenuator (BTLA), etc. ICIs targeting these receptors are being tested but success may vary across different cancer types and patient populations, so rigorous clinical trials are necessary to assess their safety and efficacy.

Moving forward, research on ICIs is expected to continue to advance, leading to the development of more effective and safer immunotherapies. Some of the areas of research that are likely to be focused on in the future include new inhibitor discovery, combination therapy, and expansion to various non-explored cancers landscape. Additionally, the effectiveness of ICIs can vary between patients, and researchers are exploring ways to differentiate responders vs. non-responders to ICIs. This may lead to the development of personalized medicine approaches, in which patients receive the treatment that is most likely to be effective for them. Moreover, identifying biomarkers that predict response to these therapies could help guide treatment decisions and lead to better outcomes for patients.

## Figures and Tables

**Table 1:** List of some studies using various mouse models to analyze the impact of ICIs in cancer.

Serial No.	Mouse Model	Therapy Studied against Target	Cancer	References
1	BLT (bone marrow, liver, and thymus) mice	PD-1	Bladder, renal, and urethra	[106,108]
2	PBMC (peripheral blood mononuclear cell) mice	PD-1/PD-L1	Lung	[109]
3	NOG (NOD/Shi-scid/IL-2Rγnul; Non-Obese Diabetic Severe Combined Immunodeficiency Interleukin-2 receptor gamma chain)	PD-1	Melanoma	[110]
4	BALB/c (Bagg Albino) mice	CTLA-4	Renal cell carcinoma	[111]
5	NSG (NOD scid gamma mouse) mice with human immune cells engrafted after birth	PD-1/CTLA-4	Breast cancer	[112]
6	NSG (NOD-scid Il2rg−/−)	PD-1/PD-L1	Hepatocellular carcinoma	[100]
7	PBL-NSG (Peripheral Blood Lymphocyte-NOD Scid Gamma mouse)	PD-1	Lung cancer	[113]
8	PDX (CD34^+^ HSC) (patient derived xenograft CD34+ hematopoietic stem cells mouse)	PD-1	Lung cancer	[114]
9	NSG-CTLA-4 (NOD scid gamma cytotoxic T lymphocyte-associated protein 4) knock-in mice	CTLA-4	Hepatocellular carcinoma	[115]
10	BALB/c-Rag2nullIl2rγnullSirpaNOD mice	PD-1	Adrenocortical cancer	[116]
11	NOG-EXL mice	PD-1	Lung cancer	[117]

**Table 2:** List of phase 1 clinical trials currently recruiting patients for ICI combined with other interventions (data retrieved from https://clinicaltrials.gov/; accessed on 22 February 2023).

Serial No.	National Clinical Trial (NCT) Number	Conditions	Interventions	Enrollment	Proposed Completion
1	NCT05303493	NSCLC Stage IV|Melanoma Stage IV|Unresectable Melanoma|Advanced Non-Small Cell Lung Cancer	Biological: Camu Camu Capsules (Camu Camu powder encapsulated (500 mg each) + ICI	45	15-Apr-27
2	NCT05430009	Liver Metastases|Non-small Cell Lung Cancer	Radiation: Liver SBRT|Drug: Pembrolizumab	12	15-Jun-26
3	NCT04290546	Squamous Cell Carcinoma of the Head and Neck|Recurrent Head and Neck Squamous Cell Carcinoma	Drug: Interleukin-15 Superagonist (N-803)|Biological: CIML NK cell Infusion|Drug: Ipilimumab	12	31-Dec-23
4	NCT05497453	Hepatocellular Carcinoma|Solid Tumor|Hepatocellular Carcinoma Non-resectable|Hepatocellular Carcinoma Recurrent|Hepatocellular Cancer|Liver Cancer|Liver, Cancer of, Non-Resectable	Drug: OTX-2002|Drug: Tyrosine kinase inhibitor One|Drug: Tyrosine kinase inhibitor Two|Drug: Checkpoint Inhibitor, Immune	190	Dec-28
5	NCT05338775	Relapsed/Refractory Multiple Myeloma	Drug: Talquetamab|Drug: Teclistamab|Drug: PD-1 Inhibitor	152	15-Oct-25
6	NCT02557321	Melanoma	Drug: PV-10|Drug: Pembrolizumab	192	Nov-24
7	NCT04187404	Adrenocortical Carcinoma|Pheochromocytoma|Paraganglioma	Biological: EO2401|Biological: Nivolumab|Biological: EO2401 and nivolumab	120	30-Dec-24
8	NCT05089370	Malignant Melanoma	Combination Product: Oral Decitabine/Cedazuridine (DEC-C) in Combination with Nivolumab	30	Jul-26
9	NCT04247165	Borderline Resectable, Locally Advanced or Metastatic Pancreatic Cancer	Drug: Gemcitabine|Drug: Nab-paclitaxel|Drug: Nivolumab|Drug: Ipilimumab|Radiation: SBRT	40	Feb-24
10	NCT05598853	Leptomeningeal Metastasis|Non-small Cell Lung Cancer Stage IV|Melanoma Stage IV	Drug: intrathecal nivolumab and intrathecal ipilimumab	26	Apr-25
11	NCT04003649	Recurrent Glioblastoma|Refractory Glioblastoma	Biological: IL13Ralpha2-specific Hinge-optimized 4-1BB-co-stimulatory CAR/Truncated CD19-expressing Autologous TN/MEM Cells|Biological: Ipilimumab|Biological: Nivolumab|Other: Quality-of-Life Assessment|Other: Questionnaire Administration	60	31-Dec-24

## Data Availability

Not applicable.
